# The Preparation of an Environmentally Friendly Novel Daidzein-Modified Lignin Phenolic Resin with High Performance and Its Application in Friction Materials

**DOI:** 10.3390/polym17010094

**Published:** 2025-01-01

**Authors:** Yufei Jia, Yimiao Zhang, Fuliang Meng, Zeyu Chen, Hongwei Fei, Dapeng Zhou, Maiyong Zhu, Xinhua Yuan

**Affiliations:** 1 School of Materials Science and Engineering, Jiangsu University, Zhenjiang 212013, China; 2 Hangmo New Materials Group Co., Ltd., Huzhou 313310, China; 3 Changzhou Haoda Technology Co., Ltd., Changzhou 213133, China

**Keywords:** biological phenolic resin, environmentally friendly materials, friction material, preparation, wear resistance, improvement of thermo-mechanical properties

## Abstract

The preparation of biological phenolic resin (PF) with green recyclable biomaterials instead of phenol is a research hotspot for solving current resource and environmental problems. In this study, on the basis of introducing lignin into the phenolic system, daidzein of a renewable resource with a rigid structure was selected to modify lignin-based phenolic resin (LPF), and the improvement of the mechanical and thermal properties of the modified phenolic resin under different substitution ratios was studied. The friction materials were prepared with a daidzein-modified lignin-based phenolic resin (D-LPF) as the matrix binder, and their effects on the mechanics and friction and wear properties of friction materials were investigated. The results show that when the proportion of daidzein replacing phenol is 12%, the highest *T*_p_ can reach 152.4 °C, and the *T*_g_ of the modified D-LPF resins is significantly higher than those of PF and LPF. The highest *T*_s_ of D-LPF is 203.3 °C, which is also significantly higher than those of PF and LPF (184.7 °C and 174.6 °C, respectively). The maximum carbon residue rate at 800 °C is 64.2% and is greatly improved compared with the 55.1% and 56.7% of PF and LPF. The bending strength and impact strength of D-LPF-matrix friction materials are obviously higher than those of PF- and LPF-matrix friction materials. The specific wear rate of D-LPF-matrix friction materials is 0.70 × 10^−4^ mm^3^/Nm, which is obviously lower than those of PF- and LPF-matrix friction materials and shows good applicational prospect as a matrix resin in friction materials.

## 1. Introduction

Phenolic resin has been under development for more than one hundred years since it was first synthesized. As an outstanding and very mature material in the field of polymer science, phenolic resin has excellent physical and chemical properties and has very important applications in various fields all over the world [1,2]. However, as the applicable fields of phenolic resin expand and the demand for it continues to grow, the environmental and resource issues it brings are becoming increasingly severe. Especially against the backdrop of the high consumption of petroleum-based products and the growing awareness of sustainable development, phenolic resin synthesized from traditional petrochemical raw materials no longer conforms to the concept of green sustainable development [3]. Therefore, the preparation of bio-based phenolic resin by promoting natural biomass materials to replace phenol and formaldehyde has become a global research hotspot in recent years.

Bio-based materials refer to the products obtained by the physical or chemical treatment of natural organic substances such as plants or animals. Common bio-based materials include wood, cellulose, straws, crop waste, aquatic plants, animal husbandry residues, and animal and vegetable oils, which come from a wide range of sources and low prices [4,5,6,7]. Bio-based materials are more environmentally friendly than petroleum-based materials, so they are often used to replace petrochemical products. Petroleum-based materials are non-biodegradable with potentially destructive effects on animals and marine organisms, and in most cases, the period from production to final decomposition is long, while most of them do not have the characteristics of renewable and natural degradation. Based on this, bio-based materials are often used to produce bioenergy, functional films or microspheres, bio-based chemicals, antioxidant activity, and other products that can replace fossil fuels [8,9,10,11,12]. The extensive use of bio-based materials helps to realize the concept of sustainable development.

**Figure 1:**
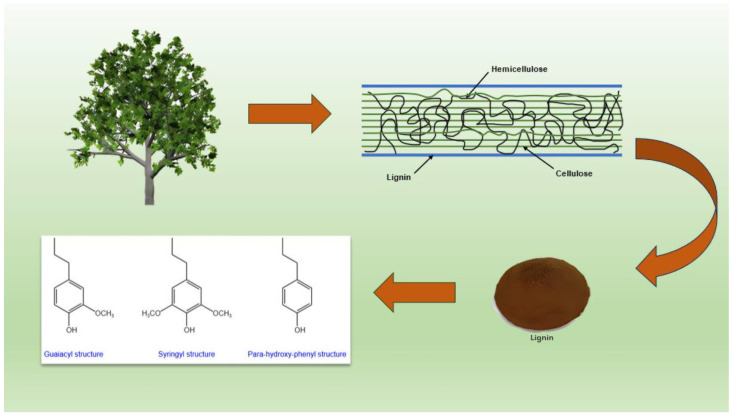
Three structural formulas of lignin.

Lignin is one of the most abundant natural aromatic substances on the earth. Because of its unique structure and chemical properties, lignin has been regarded as an important economic source of biological resources. Lignin has a natural 3D network structure, including lilac propane, p-hydroxyphenylpropane, and guaiacyl propane, as shown in Figure 1. Through the phenylpropane structural unit, they are highly cross-linked by monomer dehydrogenation polymerization to form a C-O bond and C-C bond [13]. In addition, the structure of lignin contains many aldehyde groups, alcohol hydroxyl groups, and phenolic hydroxyl groups, so lignin can provide hydroxyl and aldehyde groups at the same time during synthesizing phenolic resin, thus reducing the use of phenol and formaldehyde for protecting the environment and human health [1].

The introduction of lignin into a phenolic resin system can reduce the use of phenol and the release of formaldehyde, but it also has some adverse effects on the phenolic resin, so that its properties cannot reach the level of traditional phenolic resin. There are many kinds and sources of biological phenols in nature, which potentially facilitates further improving LPF.

**Figure 2:**
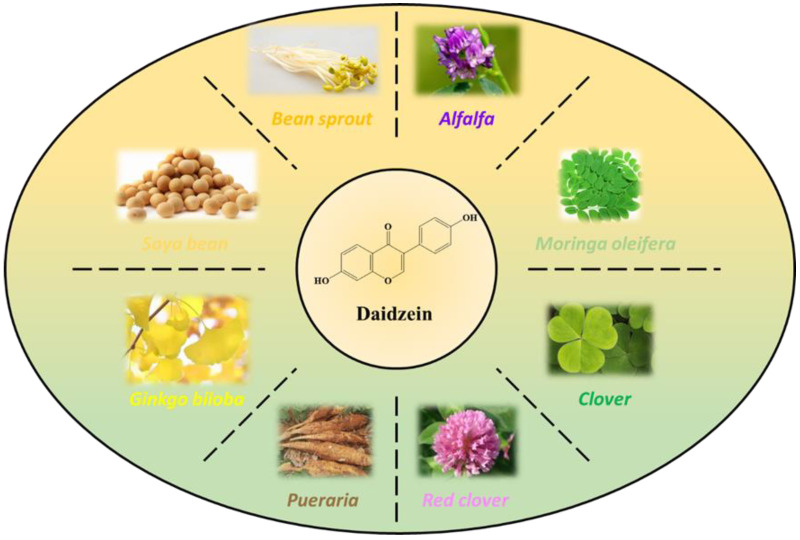
Origin and chemical structure of daidzein.

In recent years, our group partially replaced phenol with the natural biological phenol of daidzein to modify a lignin-based biological phenolic resin for further improving the comprehensive properties of LPF. Daidzein is a kind of isoflavone plant polyphenol that is widely distributed in seeds and other parts of legumes. Daidzein is the most abundant in soybean but is also abundant in bean sprouts, moringa oleifera, ginkgo, alfalfa, and clovers [14]. Daidzein has two phenolic hydroxyl groups with the special structure of rigid benzopyranone, which makes daidzein a suitable phenol source for the synthesis of various thermosetting resins [15]. There is also another method that was used to realize the biosynthesis of daidzein from naringenin [16]. The structural features of daidzein are shown in Figure 2. Relevant researchers have also carried out related research on daidzein thermosetting resin. Han et al. [14] synthesized two novel main-chain benzoxazine polymers using daidzein, aromatic/aliphatic diamine, and paraformaldehyde as raw materials. It was found that polybenzoxazine shows very high thermal stability and low flammability. The *T*_g_ value is more than 400 °C, and the heat release capacity (HRC) is less than 30 J/ (g K). Dai et al. [17] used a one-step process to synthesize high-performance flame-retardant epoxy resin (DGED) from daidzein. The cured DGED shows excellent mechanical properties and flame retardancy. At 800 °C, the residual carbon is 42.9%, and the limiting oxygen index is 31.6%.

Friction material refers to material that can produce friction when objects come into contact with each other and move with each other. These materials are usually used to control friction, reduce wear, and provide stable friction properties, etc. [18]. Friction material is an indispensable part of any moving mechanical equipment or vehicle, and its performance directly determines people’s life, health, and property safety in production and life [19]. The oldest friction material in the world is asbestos fiber-reinforced friction material, which was developed by A. Frood at the end of the 19th century and which is widely used in brake clutches, brake pads, and brake linings. However, asbestos has been proven to be harmful to the environment and human health, and its long-term use can cause diseases, such as pulmonary fibrosis and lung cancer [20,21]. Therefore, in the following century, “non-asbestos” friction materials were developed, such as metal-matrix friction materials, resin-matrix friction materials, ceramic-matrix friction materials, and new natural fiber-matrix friction materials, and the second, third, and fourth generation of new products were developed [22].

With the rapid development of science and technology, the speed of various types of vehicles and aerospace vehicles is also increasing rapidly, so the requirements for the mechanical properties, thermal properties, wear resistance, corrosion resistance, and low noise performance of friction materials are becoming higher and higher [23,24,25]. In addition, the research on friction materials has shifted from macro to micro, from qualitative to quantitative, from a single-disciplinary analysis to multi-disciplinary comprehensive research, and relevant dynamic models and micro research theories have been established. This has established a solid foundation for promoting developments, and relevant personnel have also made some progress. Zhang et al. [26] studied the effect of graphite (Gr) on the dry sliding friction performance of PF composite materials under different sliding velocities. The results indicate that the addition of Gr can effectively reduce the sensitivity of PF-matrix friction materials to the sliding velocity, thereby enhancing the stability of the coefficient of friction. Yi et al. [27] used calcined petroleum coke (CPC), talcum powder (TP), and hexagonal boron nitride (h-BN) as friction modifiers to improve the mechanical and tribological properties of PF-matrix friction composite materials. Their research indicated that various friction modifiers had different impacts on the tribological behavior of PF-matrix friction composite materials, which may be attributed to their varying degrees of adhesion to the resin matrix.

At present, resin matrix friction materials are most commonly used, which are usually composed of binders, reinforced fibers, fillers, and regulators to meet the requirements of a moderate friction coefficient, good wear resistance, low wear, and low vibration and noise. Among these, the binder plays a decisive role in the properties of friction materials. Phenolic resin is the most commonly used binder due to its excellent characteristics of low cost; good wetting ability to most components; and good thermal, mechanical, and tribological properties. However, phenolic resin is often accused of causing various braking problems, especially its poor resistance to a high temperature, sensitivity to humidity, short shelf life, and brittleness of friction materials due to its high hardness and high modulus. Because of the above defects of phenolic resin, researchers are constantly exploring new resin binders to replace phenolic resin, such as polyimide, cyanate ester, and epoxy resin. However, due to cost factors and insufficient friction properties, these resins are rarely used in commercial friction materials. Therefore, the modification of phenolic resin to improve thermal, mechanical, and tribological properties has become the research focus of this new friction material matrix.

Based on the existing problems of the phenolic resin binder in friction materials, as well as the previous research basis of our research group on friction materials, two new biological PF-matrix friction materials were prepared by using a modified phenolic resin with the best comprehensive properties as the matrix binder and compared with traditional PF-matrix and LPF-matrix friction materials. Its applicational prospect in friction materials was evaluated from the aspects of comprehensive mechanical properties and wear resistance.

## 2. Experiment

### 2.1. Experimental Materials

Phenol (99.0 wt%); a formaldehyde solution (37% wt% in H_2_O); sodium hydroxide (NaOH, 96 wt%); and hydrochloric acid (HCl, 37% *w/w*) were purchased from Sinopharm Group (Shanghai) Co., Ltd. Daidzein (96 wt%) and lignin (97 wt%) were obtained from China Aladdin Reagent (Shanghai). Distilled or deionized water was self-prepared. All the material chemicals were used as received without any purification.

### 2.2. Preparation of Phenolic Resin

#### 2.2.1. Preparation of Traditional PF and LPF

Based on a large number of previous research results from our research group and with the support of the literature, the preparation methods for PF and LPF were consistent with those of Zhang and colleagues [28].

#### 2.2.2. Preparation of Daidzein-Modified Lignin Phenolic Resin (D-LPF)

**Figure 3:**
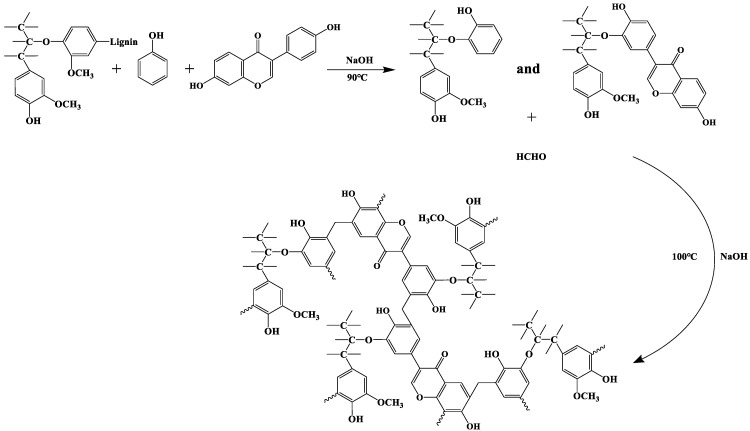
Diagram of the synthesis process of D-LPF.

On the premise of keeping the same phenolic ratio of PF and LPF, a large number of experiments were carried out at the beginning of the experiment to explore the effects of the reaction temperature, catalyst dosage, and reaction time on the properties of D-LPF. The reaction conditions were designed with temperatures of 85, 90, 95, 100, and 105 °C, with NaOH catalyst contents of 4%, 5%, 6%, 7%, and 8%, and with reaction times of 120 min, 150 min, 180 min, 210 min, and 240 min. A schematic diagram of the synthesis of D-LPF is shown in Figure 3.

Based on a large number of previous studies on reaction conditions, the specific synthesis route was determined. In the three-port flask with a reflux condenser, thermometer, and magnetic agitator, the quantitative alkali lignin, daidzein, phenol, and NaOH were added and stirred evenly, and the prepolymer was obtained after a 90 min reaction at 90 °C. After the prepolymer was naturally cooled to 60 °C, the quantitative formaldehyde was added and stirred uniformly, and the reaction was completed at 60 °C for 30 min and then at 100 °C for 210 min. After the product was naturally cooled to room temperature, neutralized to neutral with hydrochloric acid, dehydrated under vacuum conditions, and finally dehydrated in a vacuum oven at 65 °C for 48 h, D-LPF was obtained. In the above reactions, the phenolic ratio was 1.6, and the dosage of NaOH was 7% of the mass sum of phenol and daidzein. Alkali lignin accounted for 40% of the mass sum of alkali lignin, phenol, and daidzein, and the proportions of daidzein replacing phenol were 4%, 8%, 12%, 16%, and 20%. The chemicals’ mass proportions of the prepared D-LPF are shown in Table 1.

### 2.3. Preparation of PF-Matrix Friction Materials

In order to optimize the mechanical properties and wear resistance of the biological PF-matrix friction materials prepared in this experiment, according to our previous research basis of our research on resin matrix friction materials, the proportion of the binder, reinforced fiber, filler, and regulator in the friction material system was determined, and the traditional phenolic resin matrix and LPF-matrix friction materials were also prepared with the same steps and proportions.

**Figure 4:**
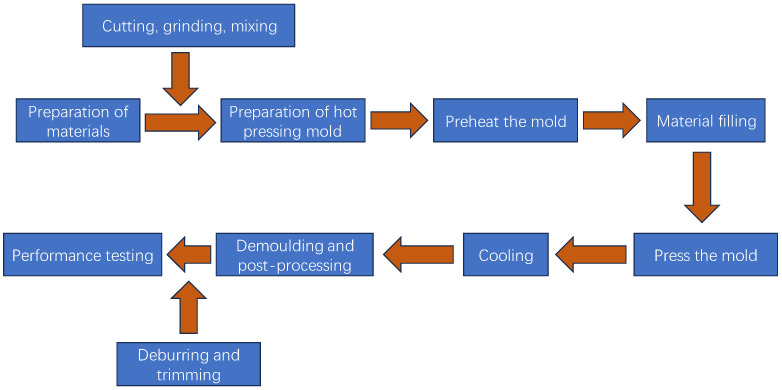
Flow chart of preparation process of friction material.

In this experiment, there was only a small number of preparation tests, so the direct mixing method was selected. The materials were mixed and placed in a customized mold, and the friction material samples were prepared by one-step hot pressing, and then, the finished products were obtained by post-heat treatment and polishing. The specific steps are shown in Figure 4.

The proportion of friction materials was based on a previous experimental study of our research group, and the proportion and function of each component are shown in Table 2. The dry direct mixing method was used in this experiment. The mixing uniformity of materials is very important to the properties of friction materials, which can avoid the formation of fiber agglomeration, so that there are no obvious irregular blocks or loose components in the system.

Friction materials were prepared by the one-step hot pressing method. The uniformly mixed material was placed in a preheated custom die and pressed at 180 °C and 10 MPa for 30 min. After the mold was cooled to room temperature, the friction material was obtained by mechanical demoulding. Then, the friction material was placed in an oven, and the friction material was prepared after heat treatment at 180 °C for 180 min at a programmed temperature. The prepared friction samples were processed by grinding and cutting to eliminate the burrs on the surface and edge of the friction materials, which may become stress concentration points in subsequent use and adversely affect their mechanical properties if they are not treated in time.

### 2.4. Performance and Characterization

#### 2.4.1. Fourier Transform Infrared Spectrometer (FTIR) of Matrix Resin

The PF, LPF, and D-LPF were dehydrated in a vacuum oven for 48 h and mixed with KBr at the ratio of 1:100 and thoroughly ground for analysis by FTIR (AVATAR360, Madison, Nicolet Instrument Corporation, Madison, WI, USA). Scanning 32 times at room temperature, the resolution and wavenumber range are 4 cm^−1^ and 500–4000 cm^−1^, respectively.

#### 2.4.2. Differential Scanning Calorimetry (DSC) of Matrix Resin

The thermal curing behavior of the resin samples was evaluated by a differential scanning calorimeter (DSC 3500 Sirius, NETZSCH Group, Gebrüder, Germany). The weighing samples were all 5 mg. At the flow rate of N_2_ at 50 mL/min, the temperature increased from room temperature to 300 °C at the rate of 10 °C/min.

#### 2.4.3. Dynamic Mechanical Analysis (DMA) of Matrix Resin

The dynamic mechanical properties of the cured resin samples were analyzed by a dynamic thermo-mechanical analyzer (DMA 242E, NETZSCH Group, Gebrüder, Germany) in single-arm mode. The samples’ temperature increased from 30 °C to 400 °C at a heating rate of 3 °C/min, the constant frequency was 5 Hz, and the sample size was about 30 mm × 5 mm × 2 mm.

#### 2.4.4. Thermogravimetric Analysis (TGA) of Matrix Resin

The thermal stability of the cured resin samples was analyzed by a comprehensive thermal analyzer (STA 449F3, NETZSCH Group, Gebrüder, Germany). The weighing samples were all 5 mg. At the flow rate of N_2_ at 50 mL/min, the temperature increased from 25 °C to 800 °C at the rate of 5 °C/min.

#### 2.4.5. Comprehensive Mechanical Properties of Friction Materials

The tensile and bending properties of the cured resin were analyzed by an electronic universal testing machine (AGS-X, Shimadzu Corporation, Tokyo, Japan). The resin powder was hot-pressed into 80 mm × 10 mm × 2 mm resin splines in a custom-made mold, and each proportional resin was tested 5 times, and then, the average value was taken.

The impact properties of the cured resin samples were tested and analyzed by a digital display plastic impact testing machine (GT-7045-MDL, GOTECH TESTING MACHINES CO., LTD., Beijing, China). The resin powder was hot-pressed into 80 mm × 10 mm × 4 mm resin splines in a custom-made mold, and each proportional resin was tested 5 times, and then, the average value was taken.

A touch screen digital Rockwell hardness tester (MHRS-150- P, Mitech CO., Ltd., Beijing, China) was used to test the hardness of the resin samples, and the average value of each proportional resin was taken after 5 times.

#### 2.4.6. Scanning Electron Microscope (SEM) of Wear Surface of Friction Material

The friction and wear properties of the resin samples were analyzed by a rotary friction tester (MS-T3001, Lanzhou Huahui Instrument Technology Co., Ltd., Lanzhou, China). Under the load of 5 N and the rotational speed of 300 r/min, the diameter of the steel ball is 4 mm, and the friction radius is 3 mm. A profilometer (KEYENCE VHX-7000N 3D, KEYENCE Corporation, Itasca, IL, USA) was used to analyze the morphology of the friction and wear resin samples and to calculate the wear amount.

#### 2.4.7. Friction and Wear Properties of Friction Materials

The fracture morphology of the resin samples was analyzed by a field emission scanning electron microscope (NOVA-NANO450, Thermo Fisher Scientific, Middleton, WI, USA).

## 3. Results and Analysis

### 3.1. FTIR of Resins

**Figure 5:**
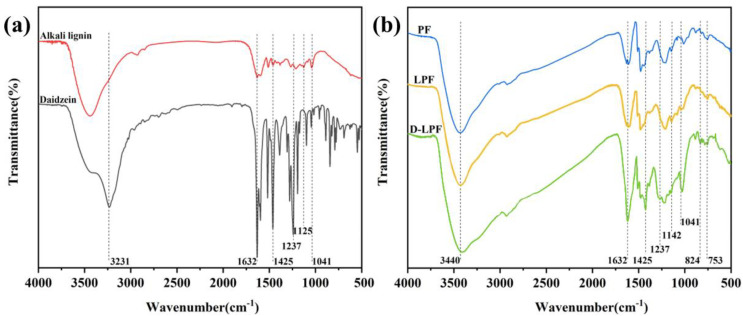
Infrared spectra of alkali lignin, daidzein (**a**), and PF, LPF, D-LPF (**b**).

The structure and functional groups of alkali lignin and daidzein were analyzed by infrared spectroscopy and are shown in Figure 5a. The structure and functional groups of PF, LPF, and D-LPF were analyzed by infrared spectroscopy and are shown in Figure 5b. From Figure 5b, it can be seen that the infrared spectra of LPF, D-LPF, and PF are roughly the same, indicating that their structures are very similar. In Figure 5a, it is shown that the hydroxyl stretching vibration peak of daidzein at 3231 cm^−1^ disappeared completely in the D-LPF pattern, while the hydroxyl stretching vibration peak appeared at 3440 cm^−1^. In addition, compared with PF and LPF, D-LPF has a strong absorption peak at 1632 cm^−1^, which is due to the C=C-C=O absorption peak of a conjugated double bond in the daidzein structure. D-LPF has a strong absorption peak at 1425 cm^−1^, which is the in-plane bending vibration absorption peak of the C-H bond. D-LPF has an absorption peak at 1237 cm^−1^, which is the absorption peak of C-O-C in the benzopyranone structure [15]. All these absorption peaks correspond to the infrared spectra of daidzein, indicating the successful introduction of daidzein. In Figure 5b, the vibrational absorption peak of the ether bond at 1142 cm^−1^ and the two peaks of 824 cm^−1^ and 753 cm^−1^ are the characteristic peaks of the *p*-position and *ortho*-substitution of hydroxyl groups on the benzene ring. In addition, some typical alkali lignin patterns are also reflected in LPF and D-LPF. Figure 5b shows a C=O stretching vibration peak of the carbonyl group at 1632 cm^−1^, and Figure 5a shows the vibration peak of a lilac ring C-O in alkali lignin at 1125 cm^−1^, but it completely disappeared in the LPF and D-LPF spectra, indicating that the structure of lilac is destroyed during the synthesis of phenolic resin by an alkali lignin reaction. LPF and D-LPF have an obvious absorption peak at 1041 cm^−1^, which is the stretching vibration peak of the C-O of the hydroxymethyl of alkali lignin [29]. However, compared with the absorption peak of alkali lignin at the corresponding position, the absorption peak of alkali lignin decreased to a certain extent, which is due to the shedding of some methoxy groups in the process of synthesis.

### 3.2. Analysis of Curing Process of Resins

**Figure 6:**
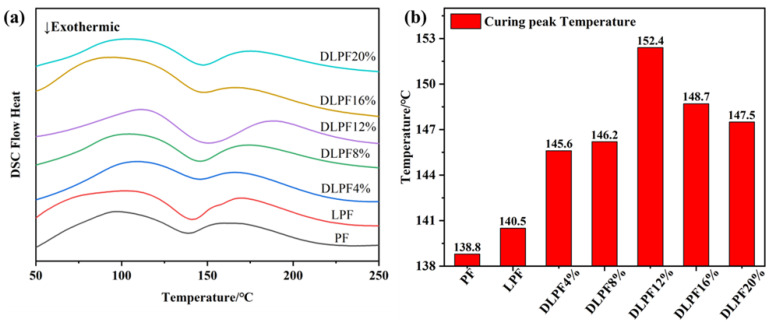
DSC spectra of PF, LPF, and D-LPF (**a**) and peak curing temperature (**b**).

The curing processes of three thermosetting phenolic resins of PF, LPF, and D-LPF were tested and analyzed by DSC. All the samples were heated from room temperature to 300 °C at the rate of 10 °C/min under an atmosphere of N_2_ with a flow rate of 50 mL/min. The test images of various types of resins are shown in Figure 6, and the related data of the initial curing temperature *T*_o_ (°C), peak curing temperature *T*_p_ (°C), end curing temperature *T*_e_ (°C), and Δ*T* (*T*_p_ − *T*_o_) are shown in Table 3. The Δ*T* represents the difference between the peek temperature and the onset temperature during the constant temperature curing reaction.

The *T*_o_ of D-LPF decreased to a certain extent compared with those of PF and LPF, but *T*_p_, *T*_e_, and Δ*T* all increased in varying degrees, indicating that the curing activity of D-LPF decreased to a certain extent. This is because the reaction activity of biophenol daidzein is not as strong as that of phenol [30], and its rigid structure and larger benzene ring can produce steric hindrance, so the curing activity of the modified resin is insufficient, and a higher temperature is needed to make up for the defect for curing completely. With an increase in the substitution rate, the *T*_p_ of D-LPF increased at first and then decreased. When the proportion of daidzein replacing phenol is 12%, the highest *T*_p_ can reach 152.4 °C, and when the proportion of daidzein replacing phenol is 4%, the lowest *T*_p_ is 145.6 °C. The difference of *T*_p_ under different phenol substitution rates is due to the different proportion of daidzein and the different degree of polymerization in different temperature ranges, thus forming intermediates with different degrees of polymerization, which may lead to a change of the resin network structure and steric hindrance. When the polymerization of the system is uniform and the steric hindrance is small, the curing reaction temperature is lower; otherwise, it may lead to an increase in the curing temperature.

### 3.3. Analysis of Thermo-Mechanical Properties of Resins

**Figure 7:**
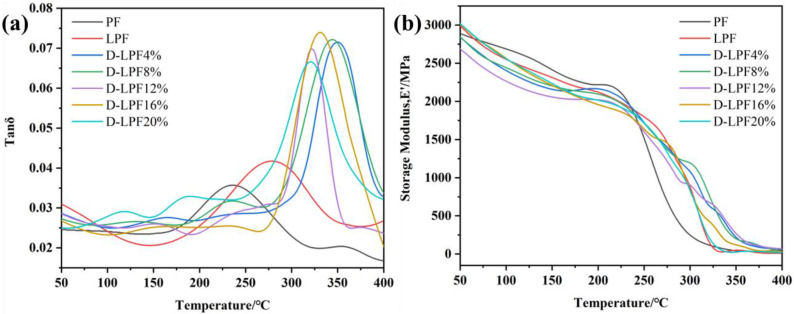
Tan *δ* signal (**a**) and energy storage modulus *E’* (**b**) of PF, LPF, and D-LPF.

The thermo-mechanical properties of D-LPF were tested and analyzed by DMA and compared with PF and LPF. The Tan *δ* and *E′* of the experimental test are shown in Figure 7. The Tan δ value represents the ratio between *E′* and *E″* of a material when it is subjected to cyclic loading.

The experimental results show that the Tan *δ* signal of the D-LPF resin is significantly narrower, and the peak amplitude is larger than those of PF and LPF, indicating that the cured D-LPF system is more uniform and has fewer internal defects [31]. When phenol is partially replaced by daidzein, the thermo-mechanical properties of the modified resin are significantly improved, and the highest *T*_g_ reached 349.8 °C, which is significantly higher than those of PF and LPF and with the respective *T*_g_ values of 236.8 °C and 279 °C. This is because the benzopyranone in daidzein can undergo a cycloaddition reaction, introducing additional cross-linking points [32], resulting in a sharp increase in the cross-linking density in D-LPF. In addition, as phenol-like substances, lignin and daidzein can form a crystalline structure in the resin and promote the formation of a cross-linked network structure, resulting in an increase in *T*_g_. However, with an increase in the proportion of daidzein replacing phenol, *T*_g_ decreased to a certain extent, which may be due to the decrease in reaction activity and incomplete curing with an increase in daidzein content. Too much of a rigid structure can hinder the migration of the polymer chain, which may enhance the damping effect and may lead to a decrease in *T*_g_.

From the thermo-mechanical properties shown in Figure 7 and Table 4, it can be seen that with the addition of daidzein, the thermal properties of the system are significantly improved, with a *T*_g_ of 346.2 °C, which is a considerable increase compared to the 236.8 °C and 279 °C of PF and LPF, respectively. This may be due to the presence of lignin and daidzein. Its inherent reactivity, similar to that of phenol, allows for the formation of a crystalline molecular framework, leading to the formation of a crystalline structure. To some extent, this promotes the formation of a cross-linked network, thereby increasing the *T*_g_. However, as the proportion of daidzein replacing phenol increases, the glass transition temperature decreases. With an increase in daidzein content, the huge rigid conjugated diphenylethylene structure hinders the synthesis and curing stages, leading to a decrease in *T*_g_. The *E*′_Max_ and *E*′_Min_ of the modified D-LPF resins are significantly higher than those of PF and LPF. This is because daidzein contains two phenolic hydroxyl groups, which can react with other phenolic hydroxyl groups in phenolic resins to form additional cross-linking points. This cross-linking increases the density of the network, thereby enhancing the thermo-mechanical properties of the material.

### 3.4. Thermal Stability Analysis of Resins

**Figure 8:**
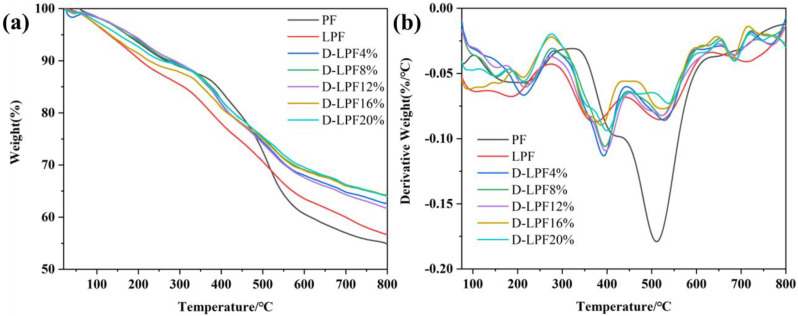
TGA (**a**) and DTG (**b**) images of PF, LPF, and D-LPF.

In order to explore the thermal stability of D-LPF, the thermal stability was tested by a comprehensive thermal analyzer and compared with PF and LPF. The tested TGA curve and DTG curve are shown in Figure 8.

The *T*_d5%_ (temperature at which a 5% weight loss occurs), *T*_d10%_ (temperature at which a 10% weight loss occurs), *T*_d30%_ (temperature at which a 30% weight loss occurs), *T*_dmax_ (temperature of maximum degradation rate), *T*_s_ (the heat resistance temperature index), and the carbon residue rate of each proportion of the samples are shown in Table 5. *T*_s_ is used to indicate the long-term service temperature of thermosetting materials, and it can also be used to characterize the thermal stability of materials. It can be obtained from the calculation in Formula (1) [33,34].

*T*_s_ = 0.49 (0.4*T*_d5%_ + 0.6*T*_d30%_) (1)

According to the test results of Table 4, it was found that the *T*_d5%_, *T*_d10%_, and *T*_d30%_ of the D-LPF resin are significantly higher than those of PF and LPF. Through the calculation of *T*_s_ by Formula (1), it was found that the *T*_s_ values of the D-LPF samples are also significantly higher than those of PF and LPF, with the respective *T*_s_ values of 184.7 °C and 174.6 °C, and the highest *T*_s_ is 203.3 °C. The carbon residue rate at 800 °C also greatly improved compared with the values of 55.1% and 56.7% of PF and LPF, and the maximum carbon residue rate at 800 °C is 64.2%. With the successful introduction of bio-phenol daidzein, the problem of the decline of thermal properties of the resin caused by lignin can be solved, and the heat resistance of the lignin–daidzein synergistic modification resin is also very excellent.

From the TGA and DTG test curves of D-LPF resins, it was found that the thermal decomposition range is similar to PF and LPF, which mainly occurs at 50–800 °C. All the curves can be roughly divided into four temperature ranges, which further illustrates the similarity of their structures. The mass loss between 50 °C and 350 °C was mainly caused by the volatilization of water, formaldehyde, and phenol residual in the resin and the breaking and removal of methyl and other small groups. The mass loss between 350 °C and 450 °C was mainly caused by the methylene decomposition of ortho-para in the resin structure. This is similar to the thermal decomposition process of various proportions of LPF resins. In this temperature range, severe degradation occurs, and their respective *T*_dmax_ appears. This is due to the cleavage and degradation of C-C and *β*-*β* bonds in its structure in this temperature range after the introduction of lignin [33], but because of the introduction of daidzein with a rigid benzopyranone structure, the *T*_dmax_ of D-LPF is significantly higher than that of LPF, indicating that daidzein helps to improve the thermal performance. The mass loss between 450 °C and 600 °C was mainly caused by the cleavage and decomposition of ether bond and methylene and the degradation of cross-linking. It can be seen from the diagram that there is an obvious degradation process of the LPF/D-LPF resins in this temperature range, which is caused by the destruction of the lignin ring network structure. From 600 °C to 800 °C, the thermal degradation of the resin tends to be stable, and the residual components form a part of the amorphous residual carbon.

### 3.5. Analysis of Comprehensive Mechanical Properties of Friction Materials

**Figure 9:**
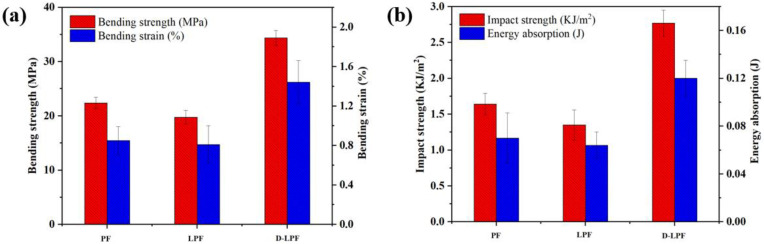
Bending strength (**a**) and impact strength (**b**) of PF-, LPF-, and D-LPF-matrix friction materials.

Friction materials are often used as brakes for vehicles or all kinds of mechanical equipment, which need to maintain the stability of structure and performance under the action of a strong braking force for a long time. Therefore, friction materials must have excellent mechanical properties. The mechanical properties of friction materials are usually expressed by bending resistance and impact resistance, and their bending strength and impact strength directly determine the service life and safety of friction materials. In order to study the bending strength and impact strength of PF-, LPF-, and D-LPF-matrix friction materials, various types of samples were tested and analyzed systematically by using an electronic universal testing machine and a plastic impact tester. The bending strength was tested by the three-point bending method, and the impact strength was tested by the single-arm beam test method. Each group of tests was carried out 5 times, and the average value was obtained. The test results are shown in Figure 9 and Table 6.

The test results show that the bending strength and impact strength of the modified phenolic resin-matrix friction materials are obviously higher than those of PF- and LPF-matrix friction materials under the same composite content and thermoforming conditions. The bending strength and impact strength of D-LPF-matrix friction materials reach 34.38 MPa and 2.77 KJ/m^2^, respectively, which are increased by 53.5% and 68.9% compared with PF-matrix friction materials and 74.0% and 105.1% compared with LPF-matrix friction materials, respectively. It was found that the mechanical properties of D-LPF-matrix friction materials are the best. The results show that the properties of a matrix resin binder have a direct effect on the friction materials. Matrix resin has a high cross-linking density and excellent mechanical properties. When the matrix resin is combined with a nitrile rubber and other reinforced fillers, the density and interfacial effects of the friction materials can be significantly enhanced, thereby endowing the material with excellent bending and impact resistance. The introduction of daidzein may introduce a certain degree of flexibility into the resin network, which helps to absorb and disperse stress, reducing the brittle fracture of the material when subjected to impact. Daidzein may improve the interfacial bonding between the resin and reinforcing fibers or fillers, possibly due to its molecular structure being more compatible with both the resin and the reinforcing materials. To sum up, D-LPF has good applicational prospect as a matrix resin of friction materials.

### 3.6. Analysis of Friction and Wear Properties of Friction Materials

**Figure 10:**
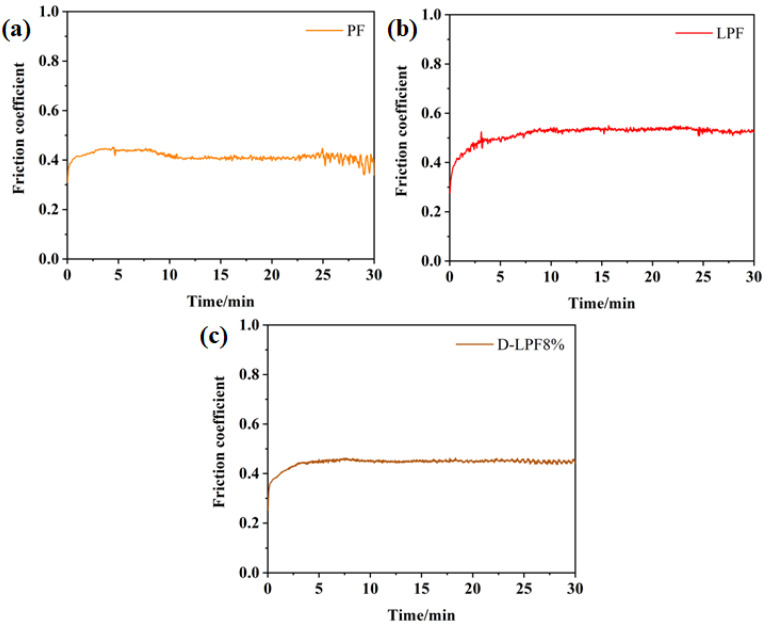
Friction coefficients of PF- (**a**), LPF- (**b**), and D-LPF- (**c**) matrix friction material.

**Figure 11:**
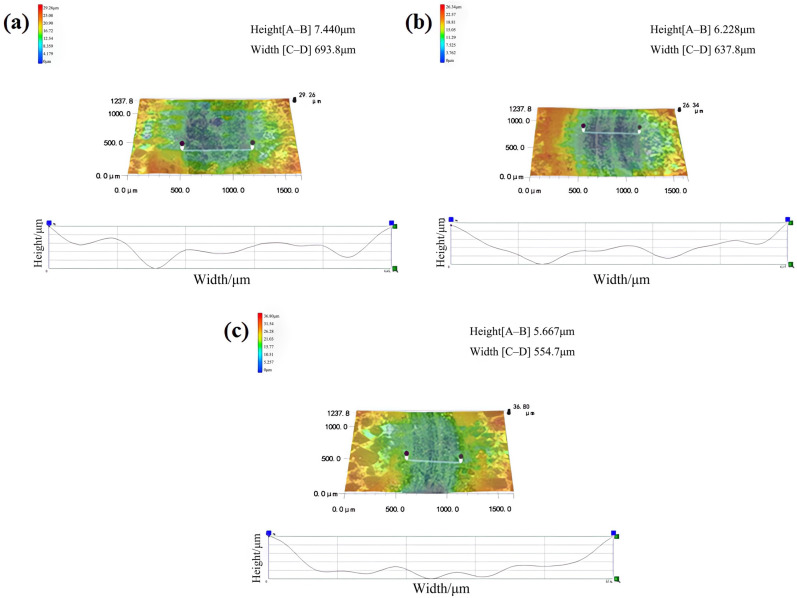
3D profiles of wear surfaces of PF- (**a**), LPF- (**b**), and D-LPF- (**c**) matrix friction materials.

The friction coefficient and wear amount are very important indexes to evaluate the properties of friction materials. In order to explore the wear resistance of friction materials based on PF, LPF, and D-LPF, the friction materials were tested by a rotary friction instrument, and the depth and width of wear marks were measured by a 3D profiler. The specific test results are shown in Figure 10 and Figure 11.

In Figure 11, the curves represent the variation curves of the height and width between two black points in the figure. Height[A–B] indicates the height difference between the two black points, and Width[C–D] indicates the width between the two black points. The scale indicates that the values increase gradually from bottom to top with the change of color.

The test results show that there are some differences in the average friction coefficients of PF-, LPF-, and D-LPF-matrix friction materials, in which the average friction coefficient of PF-matrix friction materials increases at first and then decreases. After a 25 min test, the friction coefficient fluctuated greatly and was not stable. In the whole testing process, the fluctuation period of the friction coefficient of LPF-matrix friction materials is long, but the range is not large. This is mainly due to the lack of toughness of the system after the introduction of lignin. The friction coefficient of D-LPF-matrix friction materials fluctuates little and is relatively stable, which is mainly due to the obvious increase in the heat resistance and toughness of the phenolic resin modified by resveratrol and daidzein. In the friction test, a stable carbon film forms on the surface, so it has a stable friction coefficient.

The amount of wear and the specific wear rate are calculated according to Formulas (2) and (3), and the calculation results are shown in Table 7.

The calculation formula of the wear amount Δ*V* (mm^3^) [35] is as follows:

Δ*V* = 2π*Rbh* (2)

where *R*, *b*, and *h* represent the wear radius of the resin (mm), the width of the wear mark (mm), and the depth of the wear mark (mm), respectively.

The formula for calculating the specific wear rate *K* (mm^3^/Nm) [36] is as follows:

*K* = Δ*V*/ (*LD*) (3)

where *L* and *D* represent the friction test load (N) and the sliding distance (M), respectively.

According to the calculation results of the specific wear rates in Table 7, it was found that the specific wear rate of D-LPF-matrix friction materials is 0.70 × 10^−4^ mm^3^/Nm, which is obviously lower than those of PF- and LPF-matrix friction materials. This is because the modified resin has better toughness and structural stability, which contrasts with its stable friction coefficient. The results show that the modified biological PF-matrix friction material D-LPF has good wear resistance and is a potential development direction for friction materials.

### 3.7. Analysis of Worn Surface Morphology of Friction Materials

**Figure 12:**
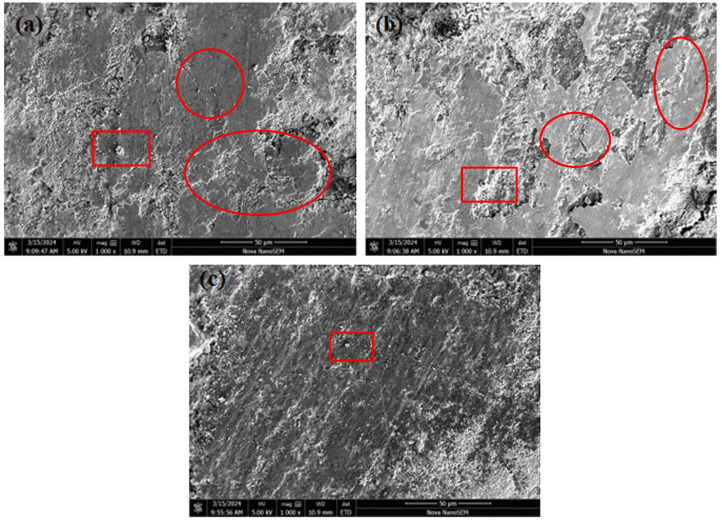
Wear surface SEM of PF- (**a**), LPF- (**b**) and D-LPF- (**c**) matrix friction materials.

SEM was used to observe the surface wear marks of PF-, LPF-, and D-LPF-resin-matrix friction materials. The test results are shown in Figure 12.

In Figure 12, the holes are marked with red rectangles, and the cracks are marked with red circles. From the SEM spectra, it was found that there are a large number of cracks on the wear surface of PF and LPF-matrix friction materials, accompanied by the shedding of granular and lamellar components. This shows that the bonding ability and mechanical properties of the matrix binder are insufficient, and the material is seriously worn when dealing with the friction of steel balls for a long time. Compared with PF- and LPF-matrix friction materials, the friction surface of D-LPF-matrix friction materials is more smooth and complete, with almost no cracks and components falling off. In addition, there are fewer pores on the sample surface, indicating that the distribution of components is uniform, which is conducive to the action of lubricating fillers on the friction surface, promoting friction heat dissipation and reducing the wear rate. This is consistent with the above test results of the matrix resin, indicating that D-LPF has the potential to be used as a matrix of friction materials.

## 4. Conclusions

Phenolic resin is a traditional thermosetting resin material, and the raw materials of phenol and formaldehyde are mostly extracted from petrochemical products. Its non-renewable and highly toxic performance and extensive use have a serious impact on human health and the natural environment. To solve this problem, on the basis of the extensive use of natural lignin, natural bio-phenol daidzein was selected to partially replace phenol, and a D-LPF-modified phenolic resin was successfully prepared. The structural functional group, curing process, thermal stability, and dynamic mechanical properties of D-LPF were systematically tested and analyzed by FTIR, DSC, DMA, and TGA, and D-LPF was compared with PF and LPF in detail. The thermo-mechanical properties and thermal stability of D-LPF are better than PF and LPF. Using the prepared D-LPF as the matrix binder of friction materials, and on the basis of previous research from our research group, a formula of D-LPF-matrix friction materials was developed. The mechanical properties, friction and wear properties, and wear surface morphology of friction materials were tested and analyzed by a universal testing machine, impact tester, rotary friction instrument, 3D profile measuring instrument, and SEM. The mechanical properties and friction properties of D-LPF-matrix friction materials are better than those of PF and LPF. To sum up, the successful preparation of D-LPF reduces the use of fossil raw materials, improves the decline of phenolic resin properties after the introduction of lignin, and makes it affords it great potential in the field of friction material matrices and sustainable bio-based resin.

## Figures and Tables

**Table 1:** Mass proportions of chemicals in the preparation of D-LPF.

Resin	Phenol	Formaldehyde	Lignin	Daidzein	NaOH
D-LPF4%	54.144	48	37.6	2.256	3.948
D-LPF8%	51.888	48	37.6	4.512	3.948
D-LPF12%	49.632	48	37.6	6.768	3.948
D-LPF16%	47.376	48	37.6	9.024	3.948
D-LPF20%	45.12	48	37.6	11.28	3.948

**Table 2:** Material ratio and function.

Component Name	Proportion	Function
Matrix resin	40%	Binder
Glass fiber powder	23%	Reinforcement
Nitrile butadiene rubber	8%	Bonding and noise reduction
Al_2_O_3_	8%	Adjust friction coefficient
MoS_2_	2%	Lubrication to reduce friction
SiO_2_	10%	Adjust friction coefficient
SiC	7%	Adjust friction coefficient
Copper powder	2%	Reinforcement and adjusting friction coefficient

**Table 3:** DSC parameters of PF, LPF, and D-LPF.

Resin Type	DSC			
	*T*_o_ (°C)	*T*_p_ (°C)	*T*_e_ (°C)	Δ*T* (*T*_p_ − *T*_o_)
PF	110.8	138.8	160.7	28.0
LPF	109.7	140.5	170.7	30.8
D-LPF4%	105.9	145.6	165.8	39.7
D-LPF8%	102.8	146.2	174.0	43.4
D-LPF12%	111.2	152.4	188.3	41.2
D-LPF16%	104.3	148.7	166.4	44.4
D-LPF20%	104.9	147.5	175.3	42.6

**Table 4:** Thermal and mechanical properties of PF, LPF, and D-LPF.

Resin Type	DMA		
	*E*′_Max_ (MPa)	*E*′_Min_ (MPa)	*T*_g_ (°C)
PF	3014.1	34.6	236.8
LPF	3185.9	48.2	279.0
D-LPF4%	3030.6	67.9	349.8
D-LPF8%	3069.8	33.7	346.2
D-LPF12%	2883.7	69.3	321.8
D-LPF16%	3209.6	50.1	330.9
D-LPF20%	3200.9	43.6	320.7

**Table 5:** Thermal stability analysis of PF, LPF, and D-LPF.

Resin Type	TGA					
	*T*_d5%_ (°C)	*T*_d10%_ (°C)	*T*_d30%_ (°C)	*T*_dmax_ (°C)	*T_s_* (°C)	Char Yield (%)
PF	171.5	265.2	514.0	511.1	184.7	55.1
LPF	129.3	206.1	507.8	372.8	174.6	56.7
D-LPF4%	183.9	276.0	555.3	392.5	199.3	62.6
D-LPF8%	174.1	281.2	575.6	395.7	203.3	64.1
D-LPF12%	185.5	287.9	552.6	397.2	198.8	61.8
D-LPF16%	132.1	226.3	569.4	387.6	193.3	64.2
D-LPF20%	152.2	257.4	587.3	400.1	202.5	64.0

**Table 6:** Mechanical property parameters of PF-, LPF-, and D-LPF-matrix friction materials.

Resin Type	Mechanical Property Test			
	Bending Strength (MPa)	Bending Strain (%)	Impact Strength (KJ/m^2^)	Impact Energy (J)
PF	22.39 ± 1.06	0.85 ± 0.14	1.64 ± 0.15	0.07 ± 0.021
LPF	19.75 ± 1.25	0.81 ± 0.19	1.35 ± 0.21	0.064 ± 0.011
D-LPF	34.38 ± 1.36	1.44 ± 0.22	2.77 ± 0.18	0.12 ± 0.015

**Table 7:** Friction and wear analysis of PF-, LPF-, and D-LPF-matrix friction materials.

Resin Type	Friction Performance Analysis	
	Average Friction Coefficient	Specific Wear Rate (mm^3^/Nm)
PF	0.4151	1.15 × 10^−4^
LPF	0.5168	0.88 × 10^−4^
D-LPF	0.4855	0.70 × 10^−4^

## Data Availability

The original contributions presented in this study are included in the article. Further inquiries can be directed to the corresponding author.
